# Changes in immune cell populations following KappaMab, lenalidomide and low‐dose dexamethasone treatment in multiple myeloma

**DOI:** 10.1002/cti2.1478

**Published:** 2023-11-30

**Authors:** Samuel E Norton, Tiffany Khong, Malarmathy Ramachandran, Andrew J Highton, Kirsten A Ward‐Hartstonge, Jake Shortt, Andrew Spencer, Roslyn A Kemp

**Affiliations:** ^1^ Nanix Limited Dunedin New Zealand; ^2^ Myeloma Research Group, Australian Centre for Blood Diseases Alfred Hospital‐Monash University Melbourne VIC Australia; ^3^ Department of Clinical Haematology and Stem Cell Transplantation Alfred Hospital Melbourne VIC Australia; ^4^ Department of Microbiology and Immunology University of Otago Dunedin New Zealand; ^5^ Monash Haematology Monash Health Clayton VIC Australia; ^6^ Blood Cancer Therapeutics Laboratory, Department of Medicine School of Clinical Sciences at Monash Health Clayton VIC Australia

**Keywords:** cytometry, dendritic cell, KappaMab, lenalidomide, myeloma, T cell

## Abstract

**Objectives:**

Lenalidomide (LEN) is used to treat multiple myeloma (MM) and shows *in vitro* synergy with KappaMab (KM), a chimeric antibody specific for Kappa Myeloma antigen, an antigen exclusively expressed on the surface of kappa‐restricted MM cells. Lenalidomide, dexamethasone (DEX) and KM control MM *via* multiple immunomodulatory mechanisms; however, there are several additional effects of the drug combination on immune cells. Lenalidomide can increase T cell and NKT cell cytotoxicity and dendritic cell (DC) activation *in vitro*. We investigated the immune cell populations in bone marrow of patients treated with KM, LEN and low‐dose DEX in kappa‐restricted relapsed/refractory MM *ex vivo* and assessed association of those changes with patient outcome.

**Methods:**

A cohort (*n* = 40) of patients with kappa‐restricted relapsed/refractory MM, treated with KM, LEN and low‐dose DEX, was analysed using a mass cytometry panel that allowed identification of immune cell subsets. Clustering analyses were used to determine significant changes in immune cell populations at time periods after treatment.

**Results:**

We found changes in five DC and 17 T‐cell populations throughout treatment. We showed an increase in activated conventional DC populations, a decrease in immature/precursor DC populations, a decrease in activated CD4 T cells and an increase in effector‐memory CD4 T cells and effector CD8 T cells, indicating an activated immune response.

**Conclusion:**

These data characterise the effects of LEN, DEX, and KM treatment on non‐target immune cells in MM. Treatment may support destruction of MM cells by both direct action and indirect mechanisms *via* immune cells.

## Graphical abstract

This research identifies dendritic cell and T‐cell populations that are modulated by KappaMab, lenalidomide and low‐dose dexamethasone treatment in MM. We show that these changes are associated with a reduction in myeloma burden. We argue that immune signatures obtained by high‐dimensional cytometry analysis from clinical trial data can be a predictor of treatment outcome.

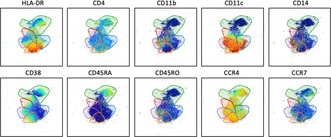

## Introduction

The immune microenvironment in multiple myeloma (MM) can be characterised by a ‘smouldering immune response’.^1^ The immunosuppressive nature of bone marrow (BM) is coupled with direct and indirect inhibitory factors produced by MM cancer cells.^2^ Macrophages and dendritic cell (DC) subsets, particularly plasmacytoid DCs (pDCs), can promote MM cell proliferation and tumor invasion by extracellular matrix remodelling, as well as providing protection from immune cell‐induced apoptosis.^3^, ^4^ Conversely, conventional (cDC1‐like), and monocyte derived pro‐inflammatory DC subsets can induce CD8 T‐cell‐mediated killing of MM cells and accumulate in the BM of MM patients compared with healthy controls.^5^ In MM patients, regulatory T cells (Tregs) can comprise up to 25% of the T‐cell compartment compared with ~15% in healthy people.^6^ T cells from MM patients have high expression of inhibitory receptors such as programmed death (PD)‐1.^1^ Together, immune responses in MM represent a complex, dysregulated milieu of suppressive and inflammatory cells and signalling molecules, and the effect of these responses on patient outcome is, therefore, difficult to study.

The combination of KappaMab (KM), lenalidomide (LEN) and low‐dose dexamethasone (DEX) is currently under evaluation as a treatment for MM. KappaMab is a chimeric IgG1 monoclonal antibody specific for Kappa Myeloma antigen (KMA), a tumor‐specific cell antigen exclusively expressed on the surface of kappa‐restricted MM cells.^7^, ^8^ Lenalidomide is an example of IMiD‐based therapy and known to have multiple immunomodulatory effects.^9^ *In vitro*, LEN upregulates the KMA target and enhances KM‐induced immune cell cytotoxicity. The specific effects of LEN on immune cell populations have been studied *in vitro*. Lenalidomide increased the expression of DC activation/maturation markers (HLA‐DR and CD86) on *in vitro*‐induced DCs from MM patient BM.^10^ Lenalidomide also increased T cell and NKT cell cytotoxicity against MM cells.^6^ In addition, LEN led to increased T cell/natural killer (NK) T‐cell interferon‐γ (IFN‐γ) production after exposure to MM cells.^11^ However, little is known about the effects of LEN, alone or in combination with KM and DEX, on total immune cell populations *in vivo*.

To better understand the *in vivo* immunological action of KM, LEN and DEX treatment, we determined the effect of treatment over time on immune cell populations in the BM of patients with MM. Bone marrow samples from 40 patients were taken pre‐treatment and at time points through five cycles of treatment and assessed by 30+ parameter mass cytometry (CyToF). Immune cell populations were defined by clustering and associated with MM cell (CD45^−^CD38^hi^) frequency as a proxy for outcome.

We demonstrate changes within major DC and T‐cell populations within 5 days after treatment initiation. These changes correlate with a reduction in MM cell frequency and provide valuable insight into the immunomodulatory action of KM, LEN and DEX treatment. By observing shifts in major immune populations from pre‐treatment over the course of treatment, we can better understand the mechanism of action of KM, LEN and DEX treatment on immune cells. Furthermore, associations between the pre‐treatment population frequencies and treatment efficacy may provide potential screening targets for use clinically.

## Results

### Identification of five dendritic cell populations following KM, LEN and DEX treatment

**Figure 1:**
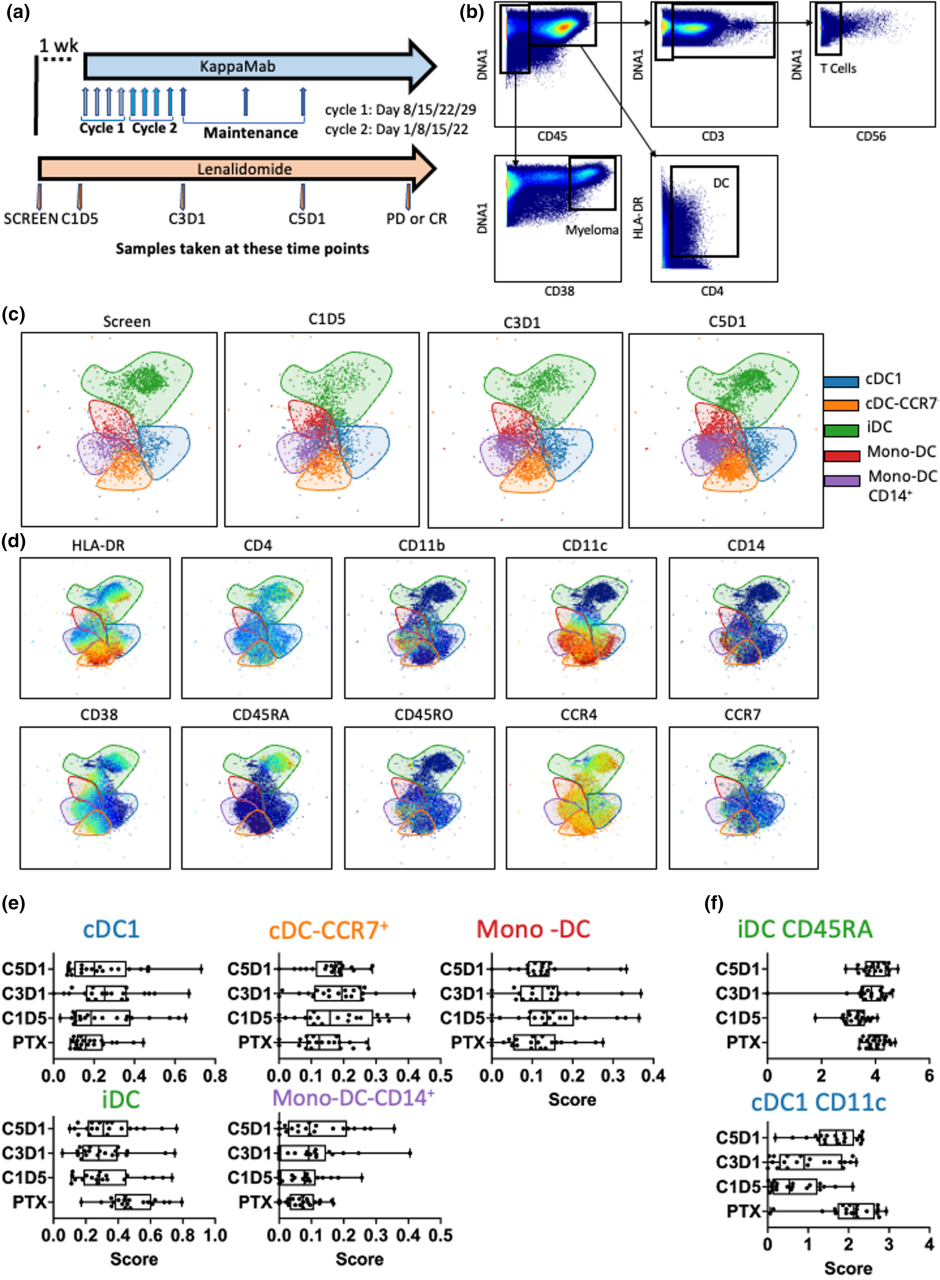
Distinct changes in dendritic cell populations following KappaMab, lenalidomide and low‐dose dexamethasone treatment. Bone marrow samples were acquired at screening and then C1D5, C3D1 and C5D1 of combination therapy and analysed by mass cytometry. **(a)** Scheme of treatment. Patients were LEN‐naive at the start of the study (SCREEN). Blue line shows KM treatment (8 blue arrows show weekly doses followed by subsequent 4‐weekly maintenance doses). Orange lines show LNE + DEX treatment as described in methods. Arrows at the bottom of the scheme show time points when samples were taken for immune analysis. **(b)** Gating and analysis strategy. Major immune cell lineages were defined by a sequential gating hierarchy. Myeloma cells were CD45^−^CD38^hi^, T cells were CD45^+^CD3^+^CD56^−^ and DCs were CD45^+^CD4^+^HLA‐DR^+^. **(c)** Identification of DC populations. Patient samples from all time points were combined and clustered using the FLOWSOM algorithm (25 total clusters). Plots show cluster ID (colour) overlaid over a UMAP dimensionality reduction plot across all four time points (SCREEN, C1D5, C3D1 and C5D1). Elbow point analysis defined 5 unique clusters: (blue) cDC1s, (orange) cDC‐CCR7^+^, (green) iDCs, (red) moDCs and (purple) moDC‐CD14^+^. **(d)** Phenotype of DC populations. Plots show relative expression (blue to green to red, low to mid to high) of each relevant DC marker (above plot) overlaid over the same UMAP as **(c)** containing events from all time points combined. Coloured outlines identify clusters defined in **c**. **(e)** Changes in DC population frequency over treatment time. Plots show SAM analysis score (relative abundance) for all 5 DC clusters at all four time points. Each dot represents a single patient per row. Box and whisker plots show median and range. **(f)** Changes in expression of markers on DC populations over treatment time. Plots show SAM analysis score (relative expression) for all iDC CD45RA expression and cDC1 CD11c expression. Each dot represents a single patient per row. Box and whisker plots show median and range. Patient data are matched across all time points. *n* = 20.

Previous reports have shown that LEN increased the expression of CD86, HLA‐DR and other activation markers on DCs and increased chemokine production from plasmacytoid DCs, but most of these data have been collected using *in vitro* assays.^10^, ^12^ To determine whether KM, LEN and DEX treatment affected immune cell populations *in vivo*, we collected BM samples from patients treated with the combination therapy for MM (Figure 1a). Bone marrow samples were taken before treatment began (pre‐treatment SCREEN), and once in each of Cycle 1, 3 and 5, as indicated.

Figure 1b shows the gating strategy for T cells, DC and myeloma cells and the analysis pathway to identify (i) clusters of immune populations, (ii) phenotype of these clusters and (iii) expression of surface markers within each cluster. We used conventional expert‐gating to define the major cell populations (T cells, CD45^+^CD3^+^CD56^−^, DCs: CD45^+^CD3^−^CD19^−^CD56^−^HLA‐DR^+^CD4^+^ and myeloma cells, CD45^−^CD38^hi^) then subsequent FLOWSOM clustering to define the downstream subsets. Supplementary figure 3 shows the frequency of the major immune cell populations (CD45^+^ cells) at all four time points.

We identified five DC populations (Figure 1c) at all four time points—SCREEN, Cycle 1 Day 5 (C1D5), Cycle 3 Day 1 (C3D1) and Cycle 5 Day 1 (C5D1). Each cluster of cells represents a distinct cell phenotype (Figure 1d). Cluster 1 (classical (c)DC1; blue) is characterised by high expression of CD11c, intermediate (relative to other DCs) expression of HLA‐DR and low expression of CD11b, CD14 and CD38. Cluster 2 (cDC‐CCR7^+^; orange) has a similar phenotype to the cDC1 cluster but with higher expression of CCR7 and CD38. cDCs can be identified by expression of either CD141 or XCR1; however, these markers were not included in the panel. Cluster 3 (immature (i)DC; green) is characterised by high expression of CD45RA and CCR4, but low expression of CD11b, CD11c, CD14 and HLA‐DR. Cluster 4 (monocytic (mono‐)DC; red) has lower expression of HLA‐DR and CD11c than the cDC subsets but higher expression of CD38 and CD11b. Cluster 5 (mono‐DC‐CD14^+^; purple) is similar to Cluster 4 but with higher expression of the monocyte markers CD14 and CD11b as well as higher CD45RO expression.

We then measured the frequency of these five populations over the course of treatment (Figure 1e) and analysed frequency of population against treatment course using SAM (Methods). Significance analysis of microarray considers the expression level of proteins and the frequencies of cells expressing proteins across multiple clusters. It identifies the feature (cluster or marker on cluster) as being a significant identifier between all the groups. The data presented therefore represent those clusters that have been shown to be significantly different across all four time points, rather than a traditional analysis comparing multiple groups to each other. cDC1 frequency increased from pre‐treatment to C3D1, whereas iDC frequency was reduced within 5 days of treatment start (pre‐treatment to C1D5). The expression of CD11c on cDC1 initially decreased and then increased during treatment, and the expression of CD45RA on iDC decreased (Figure 1f). Together, these data indicate that phenotype as well as frequency is affected by treatment.

### Identification of multiple T‐cell populations following KM, LEN and DEX treatment

**Figure 2:**
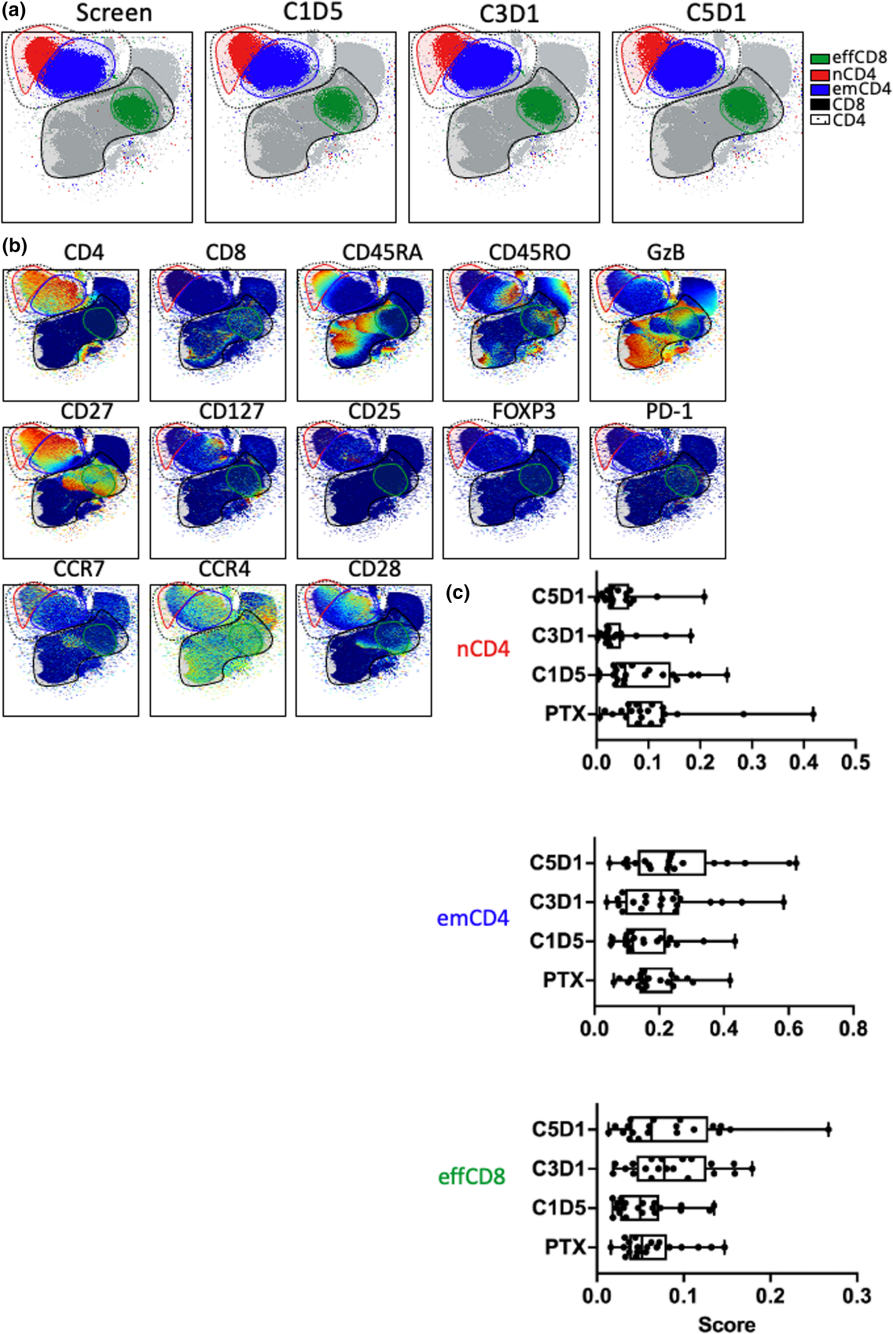
A decrease in naive T cells and an increase in effector T cells following KappaMab, lenalidomide and low‐dose dexamethasone treatment. Bone marrow samples were taken from patients with MM at SCREEN, C1D5, C3D1 and C5D1 of LEN + DEX + KM treatment and analysed by mass cytometry. **(a)** Identification of CD56^−^ T cell populations. Patient samples from all time points were combined and clustered using the FLOWSOM algorithm (100 total clusters). Plots show cluster ID (colour) overlaid over an UMAP dimensionality reduction plot across all four time points (SCREEN, C1D5, C3D1 and C5D1). Elbow point analysis defined 17 unique clusters. Three key clusters are highlighted here: (green) effector CD8 T cells, (red) naive CD4 T cells, (blue) effector memory CD4 T cells. The solid black line denotes CD8 clusters, and the dotted line denotes CD4 clusters. **(b)** Phenotype of T cell populations. Plots show relative expression (blue to green to red, low to mid to high) of each relevant T cell marker (above plot) overlaid over the same UMAP as **(a)** containing events from all time points combined. Coloured outlines identify clusters defined in **a**. **(c)** Changes in T cell population frequency over treatment time. Plots show SAM analysis score (relative abundance) for the 3 key T cell clusters at all 4 time points. Each dot represents a single patient per row. Box and whisker plots show median and range. Patient data are matched across all time points. *n* = 20.

Given the known effects of LEN on T cells *in vitro*, and the DC population heterogeneity observed in Figure 1, we analysed CD56^−^ T‐cell populations during KM, LEN and DEX treatment. Seventeen T‐cell populations were identified, the most abundant five of these are shown in Figure 2a and b shows the phenotype of all populations.

CD4 T cells are shown by the dotted line and CD8 T cells by the solid line (Figure 2a). Two CD4 T‐cell subsets and one CD8 T cells were further characterised after being identified as significant features by SAM analysis. The two CD4 T‐cell populations were differentiated by expression of CD45RA, CD45RO and CCR7, identifying a population of naive (n) CD4 T cells (CD45RA^+^ CD45RO^−^ CCR7^+^) and a population of effector memory (em) CD4 T cells (CD45RA^−^ CD45RO^+^ CCR7^−^; Figure 2b). The CD8 T‐cell population was identified as an effector (eff) T‐cell population, based on low expression of CD45RA, high expression of CD45RO, high expression of Granzyme B and low expression of CCR7. Figure 2c shows that nCD4 T cells decreased over the course of treatment, while emCD4 and effCD8 T cells both increased over the course of treatment, using SAM. Associations between DC and T‐cell populations are shown in Supplementary figure 1; these data indicate that DC populations may affect subsequent T‐cell phenotypes.

In conclusion, there was significant heterogeneity in the T‐cell populations following treatment, but an increase in effector CD4 and CD8 T cells over time. Based on these data, we then investigated the effect of the changes in immune populations on patient outcome.

### Changes in immune cell frequencies and relationship to patient outcome

**Figure 3:**
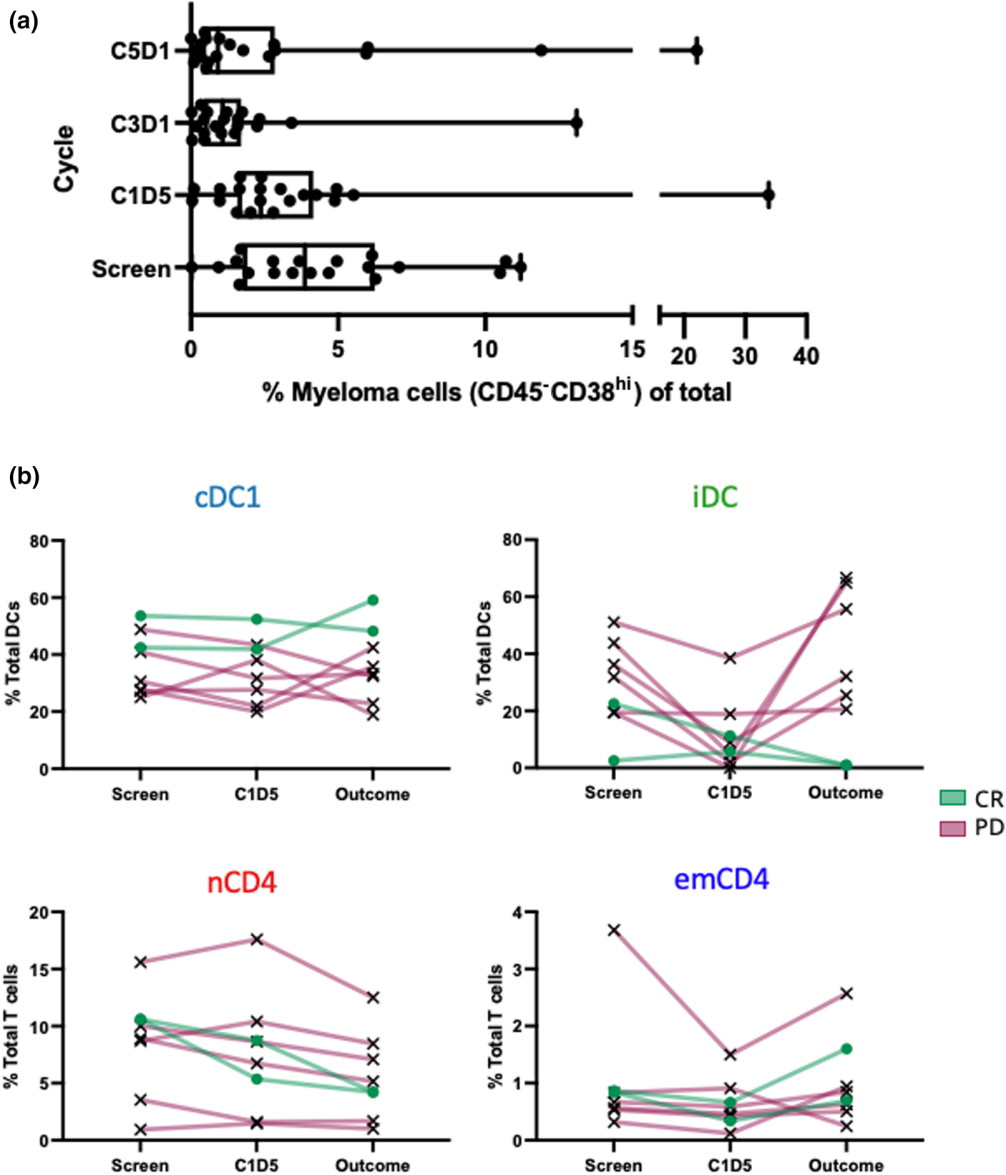
Successful treatment of multiple myeloma is associated with DC populations. Bone marrow samples were taken from patients with MM at SCREEN, C1D5, C3D1 and C5D1 of KappaMab, lenalidomide and low‐dose dexamethasone treatment and analysed by mass cytometry. **(a)** Changes in myeloma cell frequency over treatment time. The plot shows myeloma cell (CD45^−^CD38^hi^, Figure 1b) frequency at all four time points. Each dot represents a single patient per row. Box and whisker plots show median and range, and significance was determined using paired, nonparametric ANOVA; **P < 0.01. Patient data are matched across all time points. *n* = 20. **(b)** Changes in DC and T cell populations in a sub‐cohort of patients with disease progression or complete response over treatment time. Plots show cDC1, iDC, nCD4 and emCD4 frequencies (out of total DCs or T cells) for 2 patients with CR (green lines/dots) and 6 patients with PD (red lines/crosses) at SCREEN, C1D5 and Outcome (the time at which they were removed from the study after being declared in a state of CR or PD). Connected lines indicate matched data from the same patient.

The efficacy of the combination therapy is shown in Figure 3a—myeloma cells were defined to be CD45^−^CD38^hi^ (Figure 1b) and frequency measured at the same time points as immune cells. There was a statistically significant decrease in MM cell frequency at C3D1 than in SCREEN. Associations between other immune population frequencies and myeloma load are shown in Supplementary figure 2; these data show cDC1 populations negatively associated with MM burden.

We identified eight (of 40) patients who had either disease progression (red; *n* = 6, PD) or Complete Response (green/blue; *n* = 2, CR) after starting KM, LEN and DEX treatment (Figure 3b). The two patients who achieved CR occurred after C3D1 and so were removed from the study prior to C5D1. We discovered three key distinguishing CR features when looking at cluster frequency changes from pre‐treatment to outcome compared with patients who had PD. First, the two CR patients retained the highest frequency of cDC1 from pre‐treatment to outcome. Second, the two CR patients had among the lowest iDC frequency throughout. Both had no iDCs by CR. There were no differences in T‐cell populations between the CR and PD groups over time.

## Discussion

We assessed the immune complexity in MM patients receiving KM, LEN and low‐dose DEX treatment. Given the known immune effects of LEN, we hypothesised that treatment with LEN, in combination with KM and DEX, would lead to immune complexity in MM patients that was more vast than previously thought. Our analysis focussed on combinations of markers representing cell subsets, rather than individual markers, and identified several DC and T‐cell populations that were altered in frequency during the treatment course. Other cell subsets were either not altered between time points, were present at very low frequencies or represented subsets outside the focus of the manuscript. We also show preliminary data indicating that changes in such populations may indicate response to treatment in MM. We conclude that multiple cell populations in bone marrow could be a collective predictor of disease progression.

We identified five populations of DCs that were affected by KM, LEN and DEX treatment in this MM cohort. The populations of DCs that increased over time had a more mature/activated phenotype (HLA‐DR^hi^, CD45RA^−^, CD11c^hi^). Dendritic cells from MM patients have been shown to be functionally defective and/or have a decreased expression of maturation markers.^13^, ^14^ Our finding supports previous data demonstrating that LEN can increase expression of mature DC markers *in vitro* and that DC differentiation in treated patients was increased compared to those without LEN treatment.^10^ Interestingly, we found changes in populations of conventional, inflammatory and monocyte DCs, indicating that LEN, in combination with KM and DEX, has very broad effects on DCs, and that predicting responses based on simple subsets of DCs in patients may be limited.

Our study analysed changes in immune cell populations in patients treated with combination therapy—KM, LEN and DEX. We cannot conclude that LEN was responsible for all immune changes seen in our study. It is possible that individual therapies, rather than LEN alone, were responsible for some of the immune effects. For example, DEX, as a premedication for a RNA‐based vaccine in a mouse model of cancer, reduced serum cytokine levels and splenic cDC populations.^15^ RNA sequencing data of a small cohort of MM patients receiving bortezomib, cyclophosphamide and DEX revealed changes in cDC2 and moDC populations compared with healthy controls at a single time point.^16^ Multiple myeloma patients receiving bortezomib, thalidomide and DEX with or without the anti‐CD38 monoclonal antibody, daratumumab, had no changes in DC populations in the absence of daratumumab during 12 weeks of treatment.^17^

Myeloid‐derived suppressor cells (MDSCs) have been shown to be increased in patients with MM and in mouse models of MM.^18^, ^19^ Furthermore, *ex vivo* treatment of MDSCs with LEN could not overcome MDSC‐mediated T‐cell suppression.^20^ Unfortunately, MDSCs were not specifically analysed in our study. An analysis of NK cells was performed; however, there were no differences in NK populations during treatment course.

It has previously been shown that *in vitro‐*derived DCs from MM patients were less efficient at stimulating T cells than healthy DCs.^6^, ^21^ Lenalidomide increases the cytotoxicity of T cells against MM^11^, ^22^ and can potentiate IFNγ production.^11^ We saw an increase in effector T‐cell populations in patients treated with LEN, KM and DEX, as has previously been shown with LEN alone.^23^ However, we were unable to correlate this with patient outcome. Our analysis approach identifies populations of CD56^−^ T cells affected by treatment; we did not study changes in individual T‐cell functions, such as proliferation.

Based on the differences in DC populations, we assessed associations between the frequency of DC and T‐cell populations over the course of treatment. Before treatment, there was a negative association between the frequencies of iDCs and both naïve and effector‐memory CD4 T‐cell populations. This association was maintained at C1D5. Interestingly, by C1D5, there was a positive correlation between iDC frequency at pre‐treatment and effector CD8 frequency at C1D5. Conversely, there was a positive correlation between cDC1s and both CD4 populations prior to treatment. These data indicate that the presence of a more mature/activated DC population at baseline may drive CD4 T‐cell infiltration and activation. Finally, we saw a positive association between the frequency of cDC1s at C1D5 and naive CD4 T cells at the same time point, but also the effector CD4 T cells at the late time point (Cycle 5 Day 1). This suggests that while both the frequency and T‐cell associations of iDCs are lost shortly after treatment initiation, KM, LEN and DEX treatment may promote the cDC1‐CD4 T‐cell response over multiple cycles.

The bone marrow as a tumor microenvironment is likely to result in further modifications of immune cells that may be more relevant to patient outcome. Leone *et al*. identified opposing roles for DC subsets in the bone marrow of MM patients^5^—activating T cells and protecting tumor cells from being killed by T cells. Analysis of peripheral blood samples is underway and, given the accessibility of blood compared with bone marrow, determining similar changes in blood immune cell populations could be useful. Our data included a small cohort of patients that could be identified as complete responders or progressive disease. Analysis of immune cell populations in this cohort‐associated DC populations with patient outcome; however, more patients are needed to draw definitive conclusions. The strengths of our study include the cohort size and the extensive sampling timeline, allowing study of changes in immune populations over the course of KM, LEN and DEX treatment, rather than assessing treated *versus* untreated cohorts. These temporal data provide evidence for functional immune cell interactions over time. Finally, our mass cytometry and analysis approach identified novel cell populations relevant to MM.

In conclusion, we show that immune complexity in MM varies by patient, by time and by treatment. We have identified multiple immune populations involved in the disease and highlight the need to take a high‐level approach to identify immune signatures rather than defined cell populations as biomarkers of disease.

## Methods

### Patient cohort

The patients were kappa‐restricted relapsed and/or refractory MM (RRMM) with 1 to 3 prior lines of treatment and LEN naive. They were enrolled in a Phase 2 study evaluating a combination of KM, LEN and DEX. Patients received LEN 25 mg on Days 1–21 of each 28‐day cycle (35 days for cycle 1), DEX 40 mg weekly and KM (10 mg kg^−1^ IV infusion) weekly for 8 weeks then every 4 weeks as maintenance until disease progression, intolerance or withdrawal of consent. All patients also received pre‐treatment with 7 days of single‐agent LEN prior to the first dose of KM. Bone marrow aspirates were acquired at screening (SCREEN; d0), cycle 1 day 5 (C1D5), cycle 3 day 1 (C3D1) and cycle 5 day 1 (C5D1). Where possible samples were also collected at the time of achieving a complete response and/or progressive disease. The protocol was approved by the Alfred Hospital Human Research and Ethics Committee, and all patients provided written informed consent in accordance with the Declaration of Helsinki. This trial was registered on the Australian New Zealand Clinical Trials Registry at www.anzctr.org.au (ACTRN12616001164482).

### Sample collection

Bone marrow samples (4.0 mL) were collected into EDTA tubes. Mononuclear cells were isolated with Ficoll, a density gradient medium in a SepMate tube (StemCell Technologies, Canada) and processed as per the manufacturer's instructions. Enriched mononuclear cells were quantitated using TC cell counter (Bio‐Rad Laboratories, South Granville, Australia) and cryovialled at 3–5 × 10^6^ per vial. Cryovials were stored in liquid nitrogen until required.

### Mass cytometry

Cryovials were thawed at 37°C in a water bath and immediately washed. All reagents were free from heavy metal contaminants. Antibody conjugation was performed using Maxpar MCP9 Antibody Labelling Kits with Cd metal isotopes and Maxpar X8 Antibody Labelling Kits with lanthanide (Ln) Metal isotopes (Fluidigm, San Francisco, USA), following the manufacturer's instructions. Samples were barcoded using the Cell‐ID 20‐Plex Pd Barcoding Kit User Guide (Integrated Sciences), following the manufacturer's instructions. For labelling, cells were incubated with Fc block (clone, BD Pharmingen) for 20 min at 4°C. Cells were resuspended in primary antibody cocktail (Table 1) in CYTO FACS buffer (0.1% bovine serum albumin + 2 mM EDTA + 0.1% sodium azide in CyPBS; Thermo Fisher, Australia) and incubated for 40 min on ice. Cells were washed three times in CYTO FACS buffer, then vortexed. 1 mL of FOXP3 Fixation/Permeabilisation working solution (eBioscience, San Diego, USA) was added and cells incubated for 40 min at 4°C. Cells were washed in Permeabilisation Buffer (eBioscience), then incubated with normal mouse/rat serum (Sigma Aldrich, St Louis, USA) for 15 min at room temperature. Directly conjugated antibodies to intracellular antigens were added (Table 1) and incubated for 30 min at room temperature. Cells were washed twice with Permeabilisation Buffer then incubated with Iridium intercalator (Integrated Sciences) at 4°C at least overnight. Cells were acquired on a CyTOF‐Helios Mass Cytometer within 2 days.

**Table 1:** Antibodies for mass cytometry

Isotype	Antibody	Clone	Supplier
172Yb^a^	CD8	RPA‐T8	Biolegend
161Dy^a^	CD24	ML5	Biolegend
141Pr^a^	CD335	9E2FGPUR	Tonbo/Jomar
150Nd^a^	CD194	CCR4	Biolegend
173Yb^a^	CD158b (KIRDL2/L3, NKAT2)	DX27	Biolegend
175Lu^a^	CD197	CCR7	Biolegend
156Gd^a^	CD158a (KIRDL1)	LS‐C16155	Sapphire Bioscience
CD336‐PE^a^	CD336	p44‐8	BD Bioscience
CD159a‐APC^a^	CD159a	Z199	Beckman Coulter
113Cd^a^	CD3	UCHT1	Australian Biosearch
112Cd^a^	HLA‐DR	L243	Australian Biosearch
154Sm^a^	CD138 (syndecan‐1)	DL‐101	Australian Biosearch
170Er^a^	Kappa (Ig light chain K)	MHK‐49	Australian Biosearch
146Nd	IgD	IA6‐2	Millennium Science
142Nd	CD19	HIB19	Millennium Science
145Nd	CD4	RPA‐T4	Millennium Science
171Yb	CD20	2H7	Millennium Science
209Bi	CD16	3G8	Millennium Science
176Yb	CD127	A019D5	Millennium Science
167Er	CD38	HIT2	Millennium Science
149Sm	CD25	2A3	Millennium Science
155Gd	CD56	B159	Millennium Science
163Dy	CD57	HCD57	Millennium Science
160Gd	CD28	CD28.2	Millennium Science
162Dy	CD11c	Bu15	Millennium Science
158Gd	CD27	L128	Millennium Science
143Nd	CD45RA	HI100	Millennium Science
169Tm	CD304 (Neuropilin‐1)	12C2	Millennium Science
151Eu	CD14	M5E2	Millennium Science
148Nd	CD274 (PDL‐1)	29E.2A3	Millennium Science
164Dy	CD45RO	UCHL1	Millennium Science
152Sm	CD66b	80H3	Millennium Science
166Er	CD314 (NKG2D)	ON72	Millennium Science
159 Tb	CD337 (NKp30)	Z25	Millennium Science
174Yb	CD279 (PD‐1)	EH12.2H7	Millennium Science
89Y	CD45	HI30	Millennium Science
144Nd	CD11b	ICRF44	Millennium Science
Secondary staining			
168Er^a^	Anti‐PE		Biolegend
165Ho^a^	Anti‐APC		Biolegend
Intracellular staining			
147Sm^a^	FOXP3	PCH101	e‐Bio/Jomar
153Eu^a^	Granzyme B	GB11	Ebioscience

^a^ Lab conjugated antibody.

### CyToF Analysis

All CyToF data were analysed on the OMIQ platform (Dotmatics, USA). Briefly, data were de‐barcoded by manual gating and live cells (cisplatin^−^DNA_1^+^) selected for T cells (CD45^+^CD3^+^CD56^−^), DCs (CD45^+^CD3^−^CD56^−^CD19^−^CD4^+^HLA‐DR^+^) and MM cells (CD45^−^CD38^hi^) were then defined by expert gating. T cells and DC populations were then defined by FLOWSOM (self‐organising maps) clustering on markers relevant to each population type. DC clustering markers were HLA‐DR, CD45RA, CD45RO, CD11b, CD11c, CD4, CD8, CD25, PDL‐1, CCR4, CCR7, CD14, CD24, CD38 and CD16. T‐cell clustering markers were CD4, CD8, CD45RA, CD45RO, CD25, FOXP3, Granzyme‐B, CD27, CD28, CD57, CD127 and PD‐1. Twenty‐five clusters were generated for DC clustering, and elbow point validation was used to define five genuine clusters. Fifty clusters were generated for T‐cell clustering, and 17 genuine clusters were defined after elbow point validation. Uniform Manifold Approximation and Projection (UMAP) dimensionality reduction was used to display the data with the FLOWSOM clusters overlayed.

### Statistical analyses

Significance analysis of microarray (SAM) analysis^24^, ^25^ (used within OMIQ) was used to determine significant features (clusters) for both T cells and DCs between sampling points. Significance analysis of microarray is a significance determining tool developed for large gene microarrays but can be applied to any kind of complex expression data, including mass cytometry data.^26^ It provides a result that is more robust than simply assessing *P*‐values by using a T statistic or equivalent. The first step is to calculate a T statistic between test groups for each marker. The algorithm then calculates a false discovery rate, to take into account the chance of false positives based on the experimental parameters. It then uses permutation testing to determine which significant differences are more significant *versus* the expected curve, in order to rank the differences. The result is a more robust measure of significance that also indicates which differences are more likely to be biologically relevant. This ranking of significance was part of the selection process for choosing the most ‘important’ results. For the specific example of the data shown here, the algorithm highlighted which populations (or marker on a given population) explained the most difference between the populations.

GraphPad Prism (9.0; La Jolla, USA) was used for other statistical tests. Paired, nonparametric ANOVA was used to show frequency of MM cells at each time point. Nonparametric Spearman's analysis was used to determine associations both between clusters, and between clusters and MM cells (Supplementary figures 1 and 2).

## Author contributions

**Samuel E Norton:** Data curation; formal analysis; investigation; methodology; visualization; writing – original draft. **Tiffany Khong:** Conceptualization; data curation; investigation; methodology; writing – review and editing. **Malarmathy Ramachandran:** Investigation. **Andrew J Highton:** Data curation; formal analysis. **Kirsten A Ward‐Hartstonge:** Formal analysis; writing – review and editing. **Jake Shortt:** Investigation. **Andrew Spencer:** Conceptualization; project administration; resources; supervision. **Roslyn A Kemp:** Data curation; formal analysis; writing – original draft.

## Conflict of interest

The authors declare no conflict of interest.

## Supporting information


Supplementary figure 1
Supplementary figure 2
Supplementary figure 3

## Data Availability

The data that support the findings of this study are available from the corresponding author upon reasonable request.
