# Supplementary material for: Changes in immune cell populations following KappaMab, lenalidomide and low‐dose dexamethasone treatment in multiple myeloma
Source: Clin Transl Immunology. 2023 Nov 30;12(12):e1478. doi: 10.1002/cti2.1478 (PMC10688504; doi:10.1002/cti2.1478)
Supplement: Supplementary file 1 — Supplementary figure 1 Supplementary figure 2 Supplementary figure 3 [file CTI2-12-e1478-s001.pdf]

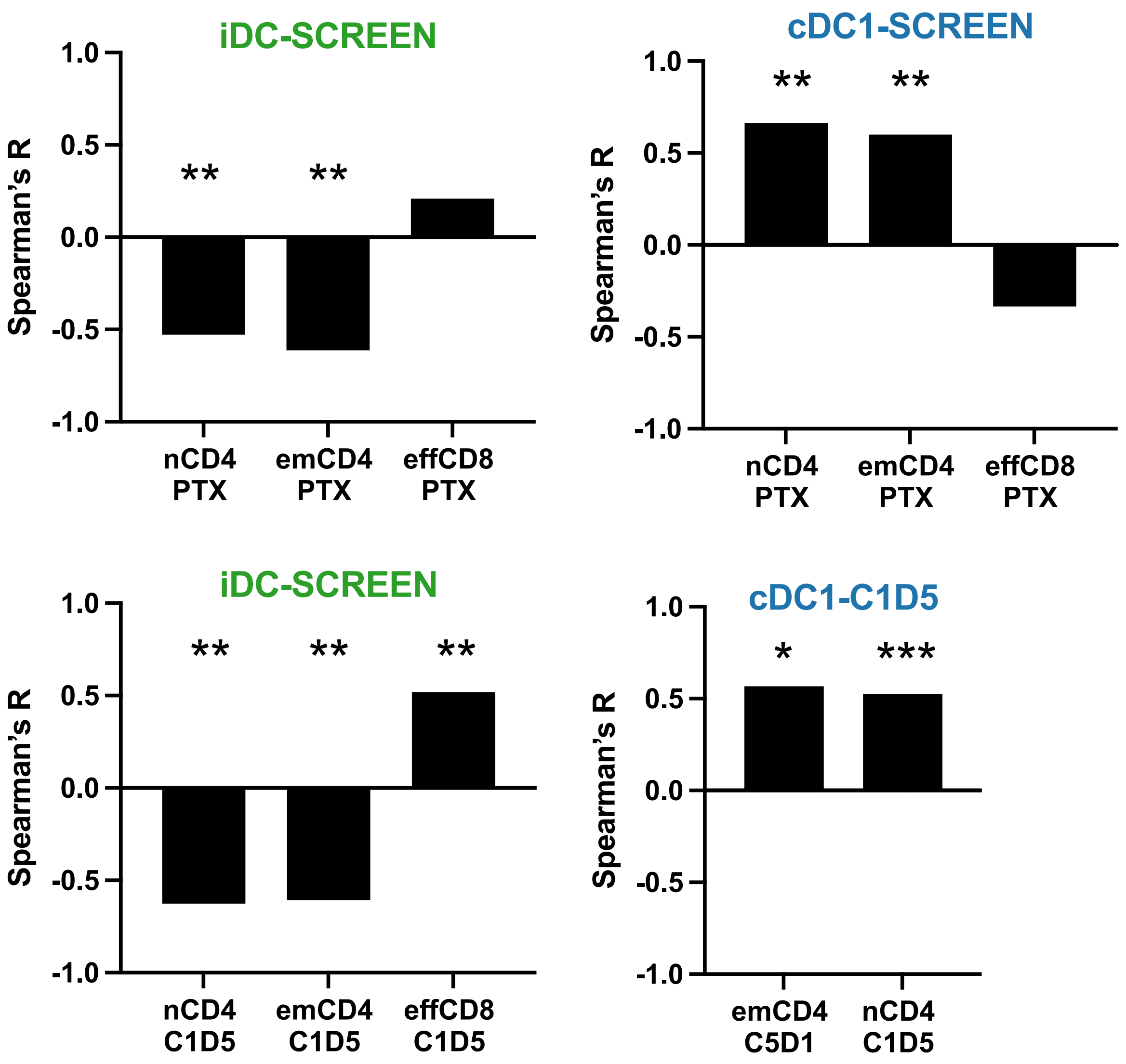

Supplementary figure 1. Associations between DC and T cell populations. Bone marrow samples were taken from patients with MM at SCREEN, C1D5, C3D1 and C5D1 of KappaMab, lenalidomide and low-dose dexamethasone treatment and analysed by mass cytometry. Plots show Spearmans R value for all significant correlations between DC and T cell populations (all other population correlations were non significant). (Top left) iDC frequnecy at screen versus all 3 T cell populations frequencies at screen. (Top Right) cDC1 frequency at screen versus all 3 T cell populations frequencies at screen. (Bottom left) iDC frequency at screen versus all 3 T cell populations frequencies at C1D5. (Bottom Right) cDC1 frequency at C1D5 versus emCD4 at C5D1 and nCD4 at C1D5. n = 20. * $P < 0.05$, ** $P < 0.01$, *** $P < 0.001$.

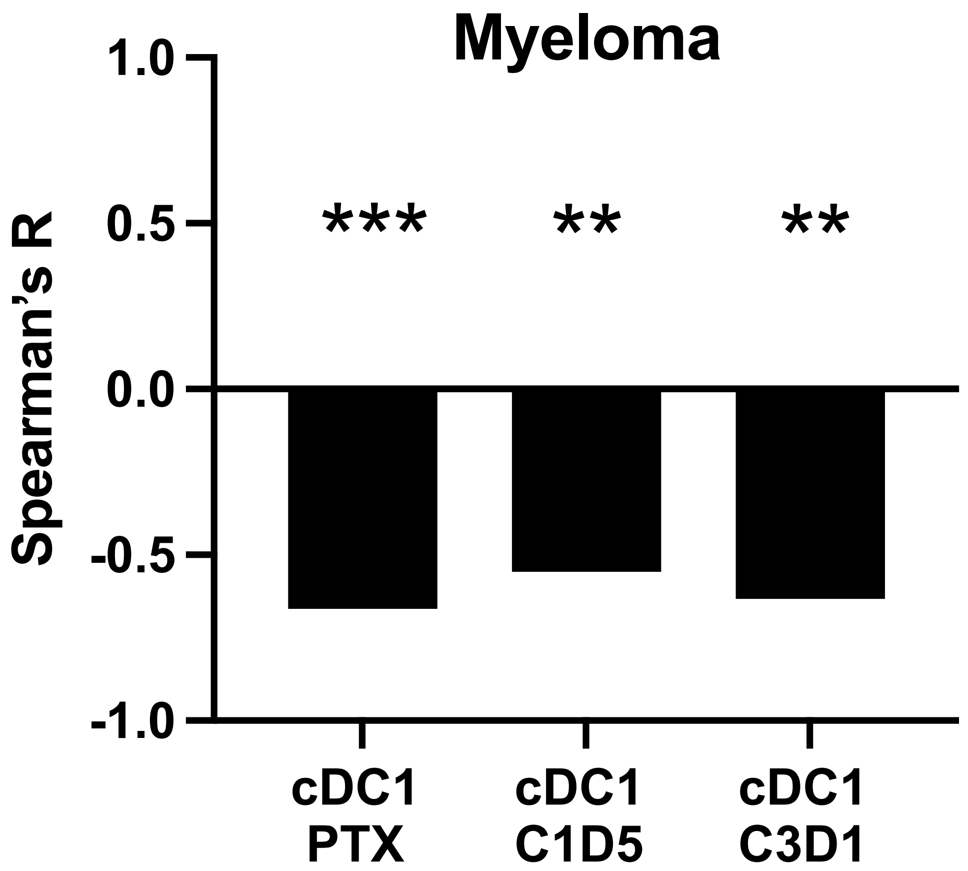

Supplementary figure 2. Associations between immune population frequencies and myeloma load. Bone marrow samples were taken from patients with MM at SCREEN, C1D5, C3D1 and C5D1 of KappaMab, lenalidomide and low-dose dexamethasone treatment and analysed by mass cytometry. Plots show Spearmans R value for all significant correlations between myeloma cell frequency and DC populations (all other immune cell population correlations with myeloma load were non significant). n = 20. ** $P < 0.01$, *** $P < 0.001$.

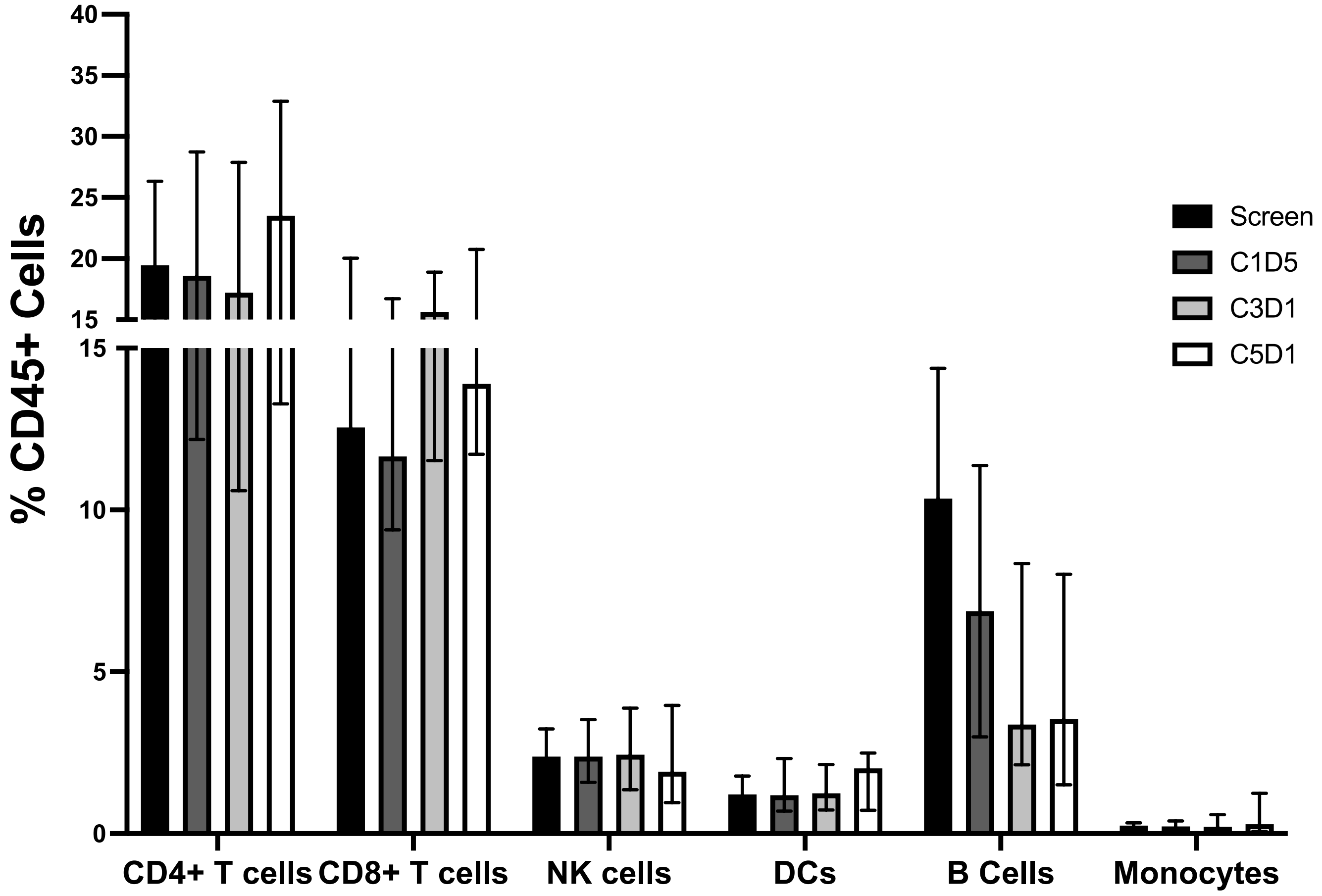

Supplementary figure 3. Immune cell (CD45+) compartment across all time points. Bone marrow samples were taken from patients with MM at SCREEN, C1D5, C3D1 and C5D1 of KappaMab, lenalidomide and low-dose dexamethasone treatment and analysed by mass cytometry. Plots show CD4$^+$ T cell, CD8$^+$ T cell, NK cell, DC, B cells and monocyte median frequency at all four time points. Error bars show interquartile range. n = 20.
